# Utilization of Waste Natural Fibers Mixed with Polylactic Acid (PLA) Bicomponent Fiber: Incorporating Kapok and Cattail Fibers for Nonwoven Medical Textile Applications

**DOI:** 10.3390/polym16010076

**Published:** 2023-12-26

**Authors:** Tanyalak Srisuk, Khanittha Charoenlarp, Piyaporn Kampeerapappun

**Affiliations:** Faculty of Textile Industries, Rajamangala University of Technology Krungthep, Bangkok 10120, Thailand; tanyalak.s@mail.rmutk.ac.th (T.S.); khanittha.c@mail.rmutk.ac.th (K.C.)

**Keywords:** sustainable, biocomposite, hot press, bicomponent fiber, medical textile

## Abstract

Disposable surgical gowns are usually made from petroleum-based synthetic fibers that do not naturally decompose, impacting the environment. A promising approach to diminish the environmental impact of disposable gowns involves utilizing natural fibers and/or bio-based synthetic fibers. In this study, composite webs from polylactic acid (PLA) bicomponent fiber and natural fibers, cattail and kapok fibers, were prepared using the hot press method. Only the sheath region of the PLA bicomponent fiber melted, acting as an adhesive that enhanced the strength and reduced the thickness of the composite web compared with its state before hot pressing. The mechanical and physical properties of these composite webs were evaluated. Composite webs created from kapok fibers displayed a creamy yellowish-white color, while those made from cattail fibers showed a light yellowish-brown color. Additionally, the addition of natural fibers endowed the composite webs with hydrophobic properties. The maximum natural fiber content, at a ratio of 30:70 (natural fiber to PLA fiber), can be incorporated while maintaining proper water vapor permeability and mechanical properties. This nonwoven material presents an alternative with the potential to replace petroleum-based surgical gowns.

## 1. Introduction

The COVID-19 pandemic has led to the global consumption of billions of face masks, contributing to significant plastic waste [1,2]. Despite polypropylene (PP) being a cost-effective primary material in personal protective equipment, concerns have arisen regarding its reliance on diminishing petroleum sources and its significant environmental impact [3]. Consequently, there has been a noticeable drive in recent years to substitute various petroleum-based products with natural, sustainable, and eco-friendly alternatives [4,5]. This pursuit has prompted an exploration of sustainable materials across industries, particularly a keen interest in natural fibers for composite materials. This interest is mainly because of the renewability, low cost, low density, and low abrasiveness of the natural materials in the polymer industry [6,7].

Numerous research studies on natural fibers for nonwoven materials have been published. For instance, milkweed has demonstrated potential for blending with bicomponent fibers and hot pressing to create a composite web, offering a potentially environmentally friendly alternative to current petroleum-based disposable surgical gowns [8]. Fages et al. [9] combined flax with polypropylene fibers using a wet-laid process, preparing them for subsequent hot press molding. The combination of wet-laid techniques with hot press molding processes enables the utilization of high natural fiber content in composite webs while maintaining acceptable properties. Ghali et al. [10] utilized the needle-punching method to blend alfa fibers with cotton, polyester, Tencel, and wool. Their observations indicated that the air permeability of nonwoven blends generally increased with the higher ratio of alfa fiber, except in the wool blend. Additionally, Möhl et al. [11] employed cellulosic fibers sourced from textile waste along with polymers derived from renewable raw materials, proposing a potential approach for upcycling textile waste.

Among natural fibers, cattail and kapok fibers have gained recent attention owing to their widespread availability and notable features, including low density and hydrophobic–oleophilic properties [12,13,14]. The cattail plant, known as the water candle, is an aquatic plant found in temperate and tropical regions across both hemispheres, thriving in various aquatic environments, such as lakes, rivers, swamps, ponds, and ditches. When reaching maturity, cattail grass exhibits a unique behavior, bursting and releasing a substantial amount of cattail fibers, posing potential risks of pollution or fires [12]. Cattail fibers, categorized as waste biomass, constitute lignocellulosic fibers composed of cellulose, hemicellulose, lignin, pectin, and wax. They demonstrate resistance to acidic environments but are susceptible to alkaline conditions [15]. Their versatile use includes applications in oil sorption [16], supercapacitors [17], and high-capacity anode material [18], and they are safe for direct human contact due to a pH of 6.7 [15]. Similarly, kapok fibers, known for their thin cell wall and large lumen, have traditionally been used as stuffing material in items like pillows and life jackets [14,19]. However, due to their brittleness and poor cohesiveness, kapok fibers have limited spinnability, which is generally considered noneconomical. Additionally, the lightweight nature of kapok fibers can pose respiratory risks to humans [20]. Annually, millions of tons of kapok waste are generated globally [21]. Several researchers have explored the utilization of kapok fibers. For instance, Wang et al. [22] pioneered eco-friendly bio-based materials using kapok/waste silk nonwoven, applicable in areas such as dressings, facial mask substrates, and packaging. Dong et al. [21] developed kapok filters for oil removal and wastewater recovery. In addition, the development of environmentally friendly bio-based materials has also been accelerated by the pursuit of sustainable practices; one such material is polylactic acid (PLA), which has been used to have a significant impact. PLA is a biodegradable and bioactive polyester derived from renewable resources such as corn, potato, sugarcane, and other carbohydrate sources [23,24]. It is commonly used in various applications, including packaging [25], biomedical devices [26], 3D printing [26], and textiles [26,27,28], due to its eco-friendly nature and biodegradability [29,30].

In this research, the focus was on developing a medical nonwoven material with specific properties such as antibacterial qualities, water repellency, light weight, and proper water vapor permeability. To achieve this, both cattail and kapok fibers, known for their low density and hydrophobic properties, were utilized. Blending them with PLA bicomponent fiber, a biodegradable polymer, was the approach taken to create the desired nonwoven material with the targeted properties for medical applications.

## 2. Materials and Methods

### 2.1. Materials

A sheath–core polylactic acid bicomponent fiber with a length of 38 mm and 2.24 dtex was purchased from Tianjin Glory Tang Textile Co., Ltd., Tianjin, China. Both the sheath and core are composed of PLA, with the sheath having a lower melting point than the core. Cattail fibers were gathered from roadside ditches in Bangkok, Thailand, while kapok fibers were locally collected from kapok trees in the same area. Seeds and other impurities in the kapok and cattail fibers were manually removed. The fibers were used in their raw state in the experiment without any chemical cleaning or pretreatment processes.

### 2.2. Preparation of Natural Fibers/Polylactic Acid Bicomponent Fiber Composite Webs

The composites of natural fibers and polylactic acid bicomponent fiber were created with blend ratios ranging from 0/100 to 70/30 by weight, employing carding and hot press techniques. A Y275A hand-operated drum-carding machine (SDL Atlas Limited, Kowloon, Hong Kong) was used to card the hand-blended fiber. This procedure was repeated 3 times to ensure that the materials were thoroughly mixed and to ensure that the fibers were distributed uniformly. After the carding process, the obtained webs exhibited a fluffy texture and had low mechanical properties, primarily attributed to the absence of chemical or physical bonding among the fibers. To improve adhesion, the carded webs were positioned within a circular mold, 11 cm in diameter, and subsequently subjected to manual hot pressing at 135 °C for 1 min, aiming to achieve a target weight of 100 g/m^2^.

### 2.3. Characterizations

SEM images of the fibers and surface of composite webs were recorded using Jeol JSM-6400 scanning electron microscope (Jeol Ltd., Tokyo, Japan). The samples were mounted on the SEM sample brass stub using double-sided adhesive tape. Prior to SEM observation, the samples underwent sputter coating with gold. Images were captured at an accelerating voltage of 15 kV.

A 204 F1 Phoenix model differential scanning calorimetry (DSC) instrument (Netzsch-Gerätebau GmbH, Selb, Germany) was used to determine specific heat capacity of both natural and bicomponent fibers. The samples in an aluminum pan were heated from room temperature to 450 °C at a rate of 10 °C/min under a nitrogen atmosphere.

The thickness values of composite webs at various ratios were measured using a 547-401A Mitutoyo digital thickness gauge (Mitutoyo Corporation, Kanagawa, Japan).

### 2.4. Properties

#### 2.4.1. Color Measurement

The CIE L*a*b* values and whiteness of composite webs were measured using the Datacolor Check II spectrophotometer (Datacolor, Trenton, NJ, USA) under D65 illuminant and a 10-degree observer angle.

#### 2.4.2. Contact Angle Measurements

The tests were performed according to BS EN 828:2013 standard using DM-CE1 contact angle meters (Kyowa Interface Science Co., Ltd., Saitama, Japan). Briefly, each droplet of deionized water, precisely 2 μL in volume, was carefully deposited onto the sample surface and measured after a 10-s interval. All measurements were conducted at a temperature of 21 ± 1 °C and a relative humidity of 65 ± 2%. Each sample underwent testing at a minimum of three different locations.

#### 2.4.3. Antibacterial Activity

The PLA, cattail fibers, and kapok fibers were evaluated against the Gram-negative bacteria *K. pneumoniae* and Gram-positive bacteria *S. aureus* following the ASTM E2149-20 standard [31]. Dynamic conditions involved 100 rpm at 37 °C for 18 h. The experiment was conducted in triplicate. After incubation, colonies were counted to determine the bacterial count. The percentage of bacterial reduction (R, %) was calculated in accordance with Equation (1):

(1) $$R=\frac{C_{0}-C}{C_{0}}\times 100$$

where

R is the percentage of reduction bacteria viability;
C_0_ is the number of bacteria colonies at contact time = 0 h;
C is the number of bacteria colonies after contact time.

#### 2.4.4. Mechanical Test

The mechanical properties were evaluated using an M350-5AT Testometric universal testing machine (Testometric Company Ltd., Rochdale, UK) following an adapted ISO 9073-3 standard. The tests were conducted at a crosshead speed of 5 mm/min with a load cell capacity of 50 kN. Rectangular specimens, sized at 10 × 60 mm, were cut using compression molded plates for the tests with a gauge length of 40 mm. At least five specimens were assessed within each composite formulation group. The mean and standard deviation for the tested samples were calculated and reported.

#### 2.4.5. Water Vapor Permeability Analysis

Water vapor permeability was assessed using the evaporative dish method in accordance with the BS 7209 standard [32]. The test involved securing the test specimen over the open mouth of a dish filled with water and measuring the initial weight of the cup. After a 6-h period, the weight was recorded again to assess any changes. Water vapor permeability (WVP) was calculated (Equation (2)).

(2) $$\mathrm{Water}\;\mathrm{vapor}\;\mathrm{permeability}\left(\mathrm{WVP}\right)=\frac{24\times M}{A\times T}$$

where

M is the loss in mass (g) of water vapor through the fabric specimen;
A is the internal area of the dish (m^2^);
T is the time between weighing (h).

### 2.5. Statistical Analysis

The experimental design involved two types of natural fiber and eight different ratios of PLA to natural fibers. The results were analyzed using one-way analysis of variance (ANOVA). The data are presented as the mean ± SD of each treatment. Mean values were calculated according to Duncan’s multiple range tests (*p* < 0.05).

## 3. Results and Discussion

### 3.1. Characterization of Fibers

**Figure 1:**
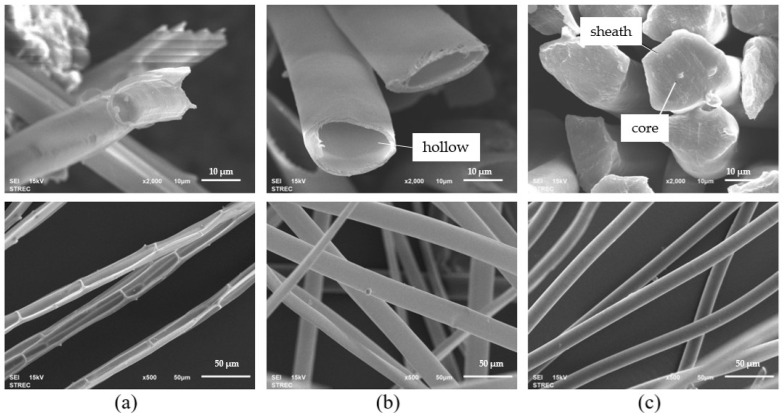
SEM images of cross-sectional (2000× magnification) and longitudinal (500× magnification) (**a**) cattail fiber, (**b**) kapok fiber, and (**c**) PLA bicomponent fiber.

Waste natural fibers, including cattail and kapok fibers, have distinct characteristics. Cattail fibers feature a multicavity structure, bamboo shape, and an average length of 10 mm, while kapok fibers have an average length of 20 mm and a hollow lumen structure (Figure 1a,b). The sheath–core PLA bicomponent fiber, depicted in Figure 1c, has a diameter of approximately 20 μm, with the sheath measuring around 2 μm in thickness.

The mechanical properties of composite materials are influenced by the length of the fibers and the fiber content [33]. Because cattail fibers are shorter in length and thinner in diameter than kapok fibers, these differences could have a significant impact on the mechanical properties of the composite webs. Kapok fibers, characterized by their thin-wall hollow structure, are filled with up to 80% air by volume and coated with hydrophobic wax, imparting hydrophobic properties and good moisture vapor permeability. Similarly, cattail fibers also possess a wax-coated surface that imparts hydrophobic characteristics. Moreover, the highly hollow nature of kapok fibers compared with cattail fibers may affect the properties of the composite webs.

**Figure 2:**
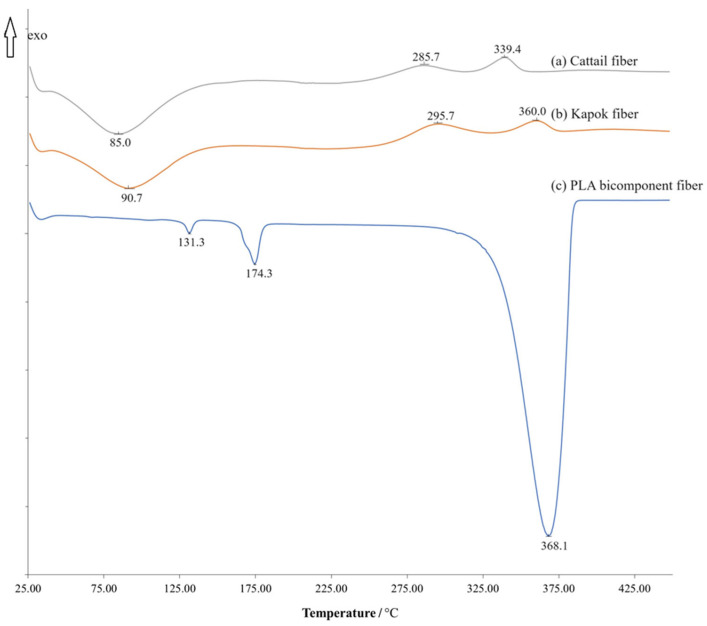
DSC thermogram for (a) cattail fiber, (b) kapok fiber, and (c) PLA bicomponent fiber.

DSC thermograms for both kapok fiber and cattail fiber exhibited similarity (Figure 2a,b). The results from the thermograms suggested that the initial weight loss occurred below 100 °C, attributed to the presence of water in the fiber [34]. The first exothermic peak appeared around 280–310 °C, indicating the degradation of cellulose. The second exothermic peak at 330–370 °C was likely associated with the oxidation of the char [35,36]. The used PLA bicomponent fiber exhibited two melting peaks at 131.3 °C and 174.3 °C. It is worth noting that the degradation temperature of PLA fell within the range of 330–380 °C (Figure 2c). These findings were similar to those of Kervran et al. [37], in which PLA degraded in one step at around 350 °C.

From the literature review, it was indicated that both cattail and kapok fibers exhibit natural antibacterial activity due to their high lignin content [38,39,40]. Cattail fiber contains an approximate lignin content of 16.5% [30], while kapok fiber exhibits a lignin content ranging from 13% to 22% [39]. In this study, the antibacterial efficacy of these fibers was evaluated according to the ASTM E2149 standard against *S. aureus* and *K. pneumoniae*. The results are presented in Table 1.

It appears that all the tested fibers did not exhibit antibacterial activity against *K. pneumoniae*. However, they showed a limited antibacterial effect against *S. aureus*. Kapok fibers demonstrated the most substantial reduction in bacterial activity when compared with both cattail and PLA bicomponent fibers. The antibacterial properties of the composite web samples were not tested since all tested fibers displayed minimal antibacterial activity.

### 3.2. Characterization of Composite Webs

In this study, the composite webs were subjected to testing for thickness, color, morphology, mechanical properties, water contact angle, and water vapor permeability after the hot pressing process.

#### 3.2.1. Thickness

The thickness values of the composite webs are presented in Table 2.

As shown in Table 2, the increase in both cattail and kapok fiber content led to an augmentation in the thickness of the composite webs. This outcome can be attributed to the notably low density of both cattail and kapok fibers. In nonwoven materials determined by weight (g/m^2^), the incorporation of these fibers with the same weight resulted in thicker nonwoven structures due to their low density. These observations are consistent with a previous study involving poly(lactic acid)/poly(butylene succinate) (PLA/PBS) fiber and milkweed fiber. In that study, it was noted that increasing the ratio of milkweed fiber resulted in enhanced thickness in the composite material [8]. Additionally, when comparing PLA: kapok fiber composite webs to PLA: cattail fiber composite webs at the same ratio, the composite webs from PLA: kapok fiber exhibited greater thickness. Kapok fiber holds the distinction of being the lightest natural fiber globally. Research conducted by Mwaikambo [41] and Sekar et al. [42] highlighted that the bulk density of kapok fiber stands at 0.38 g/cm³, whereas cattail fiber exhibits a density of 0.62 g/cm³ [43].

#### 3.2.2. Color Measurement

**Figure 3:**
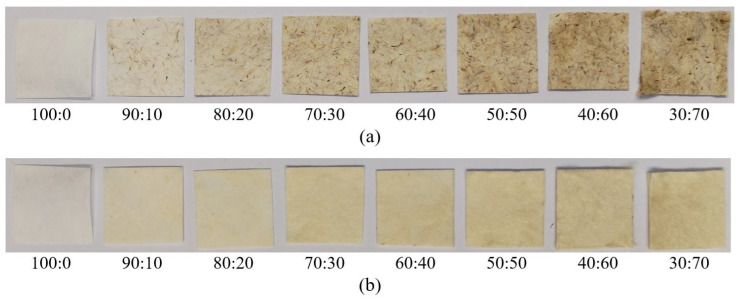
Appearance of the PLA composite with natural fiber at different ratios: (**a**) PLA: cattail fiber; (**b**) PLA: kapok fiber.

The appearance of composite webs is depicted in Figure 3, while measurements for the whiteness index and CIE L*a*b* values of these composite webs were recorded and are presented in Table 3.

The whiteness index tended to decrease with an increase in the content of natural fibers added to the PLA fiber. Additionally, the whiteness index of composite webs made from cattail fiber was lower than that of kapok fiber at the same ratio (Table 3). The color differentiation between kapok and cattail fibers was commonly observed, with kapok fibers typically appearing creamy yellowish-white, while cattail fibers tended to have a light yellowish-brown shade.

The CIE L*a*b* system comprises three values: L* (white-black), a* (red-green), and b* (yellow-blue). Higher L* values indicate a whiter appearance, while higher a* and b* values suggest more reddish and yellowish tones, respectively. With an increased natural fiber content in both cattail and kapok fibers, there was a decrease in the L* value, indicating a darker hue, and an increase in the b* value, suggesting a more yellowish coloration. Furthermore, the a* values of composite webs showed a slight increase with higher natural fiber content, indicating a tendency toward a more reddish hue in these webs.

Natural fibers inherently possess their own natural color. In this research, both natural fibers were used without pretreatment, thereby retaining their original natural color. Higher natural fiber content resulted in a more pronounced expression of their inherent colors. The obtained results showed a similar trend to the research conducted by Mula et al. [8], who used milkweed fiber mixed with bio-based bicomponent fiber. An increase in the proportion of milkweed fiber led to a yellower color in the composite. Dolza et al. [44] found that the addition of hemp fiber to virgin bio-based poly(butylene succinate-co-adipate) resulted in a decrease in L*, while the a* and b* values increased.

#### 3.2.3. Morphology of Composite Webs

**Figure 4:**
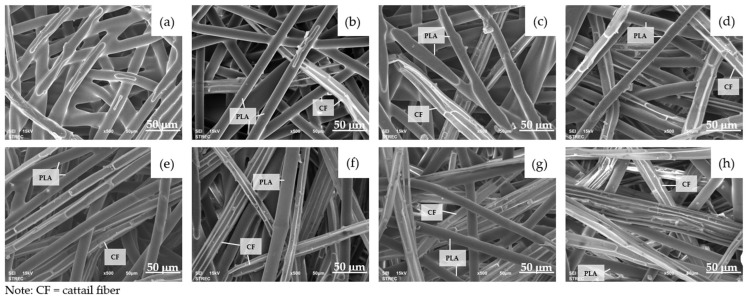
SEM images of PLA: cattail fiber composite webs at ×500 magnification: (**a**) 100:0; (**b**) 90:10; (**c**) 80:20; (**d**) 70:30; (**e**) 60:40; (**f**) 50:50; (**g**) 40:60; (**h**) 30:70.

**Figure 5:**
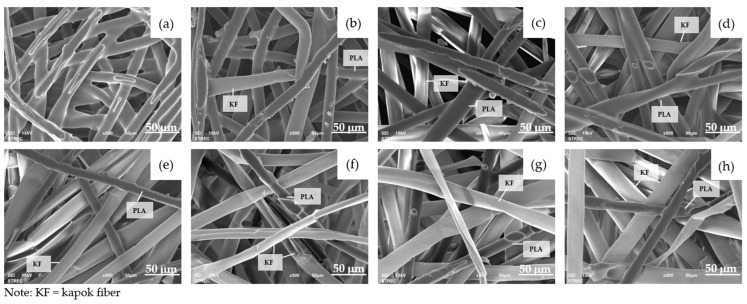
SEM images of PLA: kapok fiber composite webs at ×500 magnification: (**a**) 100:0; (**b**) 90:10; (**c**) 80:20; (**d**) 70:30; (**e**) 60:40; (**f**) 50:50; (**g**) 40:60; (**h**) 30:70.

During the hot press process at a temperature of 135 °C, the bicomponent fibers experienced partial melting, which subsequently led to the adhesion of natural fibers and bicomponent fibers together, as depicted in Figure 4 and Figure 5.

At this specific hot press temperature, the core of the PLA bicomponent remained solid, preserving the fibrous structure instead of forming a film-like structure. Meanwhile, the molten PLA sheath possessed adhesive properties, enabling it to adhere to neighboring fibers. As the proportion of natural fiber increased, the presence of PLA bicomponent fibers diminished (Figure 4 and Figure 5). When the natural fibers ratio exceeded 50%, it became challenging for the PLA to retain both natural fibers. Consequently, the composite webs might exhibit the fluffiness characteristic of natural fibers at this stage (Figure 3). This was consistent with the findings from Mula et al. [8], which similarly suggested that milkweed content should not exceed 50% due to lower mechanical properties. Jamat et al. [45] developed a kapok-fiber-reinforced polyvinyl alcohol biocomposite and found that the tensile strength and elastic modulus increased with up to 30% kapok fiber content but decreased when the kapok fiber content reached 40%. Additionally, Lee et al. [46] found that incorporating 60 wt% of silk fiber resulted in decreased flexural properties in the biocomposite. This decline was attributed to inadequate filling of the molten poly(butylene succinate) matrix around the silk fiber during the composite process.

#### 3.2.4. Mechanical Test

**Figure 6:**
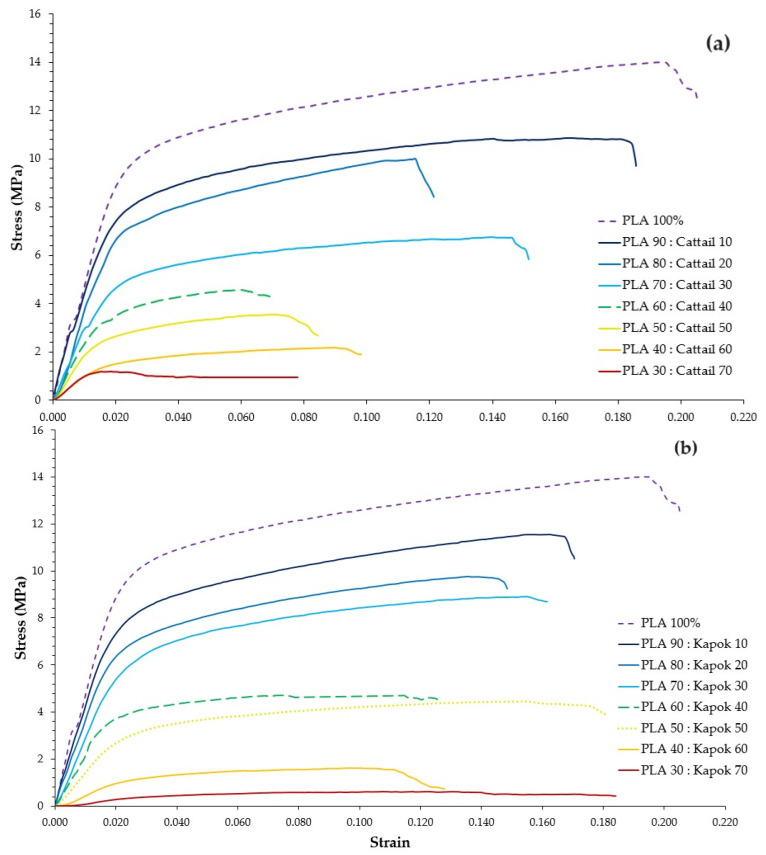
Stress–strain curves of composite webs comprising PLA bicomponent fiber mixed with (**a**) cattail fiber and (**b**) kapok fiber.

The mechanical properties of the composite webs were tested, and the data are presented in Table 4. Furthermore, the stress–strain curves of these composite webs are displayed in Figure 6.

Higher natural fiber content in the PLA bicomponent fiber composite webs resulted in reduced ultimate load, Young’s modulus, yield strength, and yield strain (Table 4). At the same natural fiber level, composite webs with kapok fiber generally exhibited better ultimate load than those mixed with cattail fiber, except in the case of PLA: cattail fiber at a 30:70 ratio. This could be attributed to the longer fiber length of kapok fiber, leading to improved interfacial bonding properties. As noted by Du et al. [47], long glass fiber composite materials tended to exhibit superior mechanical properties compared with short glass fiber composites. At a 30:70 ratio of PLA: kapok fiber, the lower density of the kapok fibers compared with cattail fibers resulted in a higher fiber content within the composite webs. Consequently, the PLA bicomponent fibers may struggle to effectively bind all the fibers, leading to significantly reduced mechanical properties.

In our study, only the sheath of the bicomponent fibers melted and acted as an adhesive to bind the natural fibers. However, a high ratio of natural fibers coupled with a low density resulted in the adhesive being unable to secure all natural fibers, which subsequently led to reduced mechanical properties (Table 4). Composite webs containing over 40% natural fibers exhibited diminished mechanical properties, a trend consistent with the findings of Mula et al.’s study [8]. In addition, according to the EN 13795-1 standard [48], the tensile strength requirement for protective clothing in the dry state is equal to or greater than 20 N. In this context, when the natural fiber ratio equaled or fell below 30, the material demonstrated an ultimate load higher than this specified standard.

#### 3.2.5. Water Contact Angle Measurement

**Figure 7:**
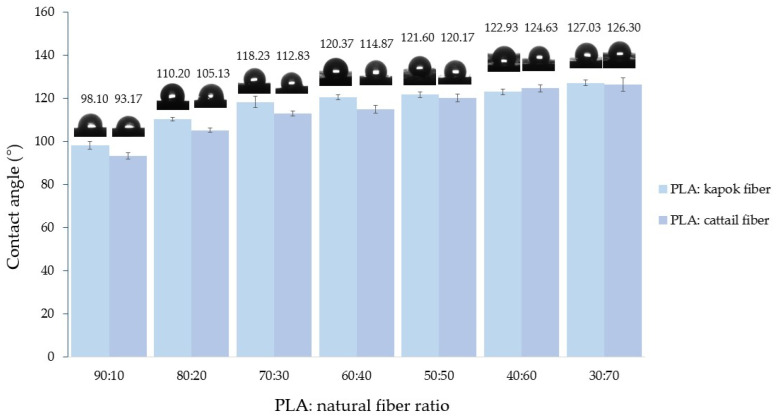
Water contact angle of composite webs.

Surgical gowns should repel blood, body fluids, and other contaminants to ensure that these substances do not penetrate the gown. This characteristic helps maintain the sterility of the gown and prevents the transmission of infectious agents between the medical professional and the patient during surgical procedures. The contact angle of a water drop on the composite webs is illustrated in Figure 7.

Contact angle values range between 0° and 180°. A contact angle of 0° corresponds to complete wetting, while an angle of 180° indicates nonwetting. A material is considered superhydrophobic if the contact angle is greater than 150°.

The PLA bicomponent web could not detect water contact angles, as it absorbed water immediately. However, the water contact angle increased with a higher natural fiber content in composite webs, as depicted in Figure 7. Ranging from 98° to 127°, the water contact angle of composite webs highlights their hydrophobic nature. This phenomenon was attributed to the surface wax present in both cattail and kapok fibers, contributing to their hydrophobic and oleophilic properties [49,50,51]. Research by Abdullah et al. [52] reported a wax content of approximately 3% in kapok fibers, while Draman et al. [35] noted a higher content of 5.51%. Based on Soxhlet extraction, Wu et al. [43] reported that wax on the surface of cattail fibers accounted for 11.5%. When comparing both natural fibers at the same ratio, cattail fibers in composite webs exhibited a slightly lower water contact angle than kapok fibers. This difference may be attributed to the lower density of kapok fibers. Consequently, at an equivalent weight, kapok fiber possessed a higher volume of fibers compared with cattail fiber. The results indicated that blending natural fibers with PLA bicomponent fibers aided in providing better protection against blood and body fluids compared with using only PLA bicomponent fiber webs.

#### 3.2.6. Water Vapor Permeability Analysis

**Figure 8:**
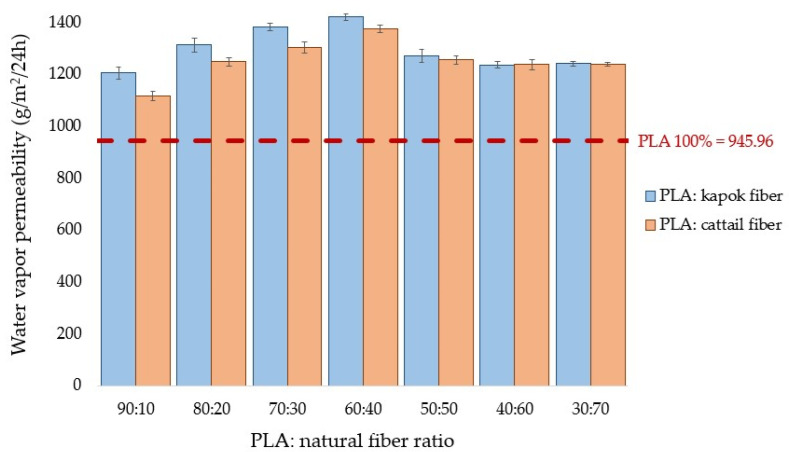
Water vapor permeability values of the composite webs.

Breathability is the ability of a textile to allow water vapor from the body through it while preventing liquid from entering from the outside [53]. This characteristic plays a crucial role in determining comfort, often assessed through the measurement of water vapor transmission rate. The water vapor permeability of the composite webs was tested, and the results are presented in Figure 8.

Incorporating natural fibers into the composite webs enhanced their water vapor permeability compared with 100% PLA bicomponent webs. Interestingly, composite webs made from cattail fibers exhibited lower water vapor permeability than those crafted from kapok fibers at identical ratios. Several factors influence water vapor permeability, including sample thickness; thicker samples increase the distance and time needed for water vapor to travel, resulting in reduced permeability. Despite composite webs from the kapok fibers being slightly thicker than those from cattail fibers at the same ratio, the significant lumen present in kapok fibers contributes to their higher water vapor permeability [54].

The findings from Kannekens [55] and Behera and Arora [53] allowed the classification of the water vapor permeability rates of breathable surgical gowns at 20 °C into different categories: moderate (360 g/m^2^/24 h), high (1392 g/m^2^/24 h), and very high (2400 g/m^2^/24 h). All composite webs showed moderate rates, ranging from 945–1384 g/m^2^/24h, except for the PLA: kapok fiber at 60:40, which exhibited a high water vapor permeability rate of 1421 g/m^2^/24 h. There might be a need in future work to reduce the thickness of composite webs to enhance water vapor permeability.

## 4. Conclusions

Composite webs comprising polylactic acid bicomponent fibers and waste natural fibers were successfully created, as outlined previously. SEM images confirmed that the composite web maintained a fibrous structure, retaining its permeability properties rather than forming a film-like structure. Evaluation of mechanical and physical properties revealed distinct color characteristics—creamy yellowish-white for kapok-fiber-based webs and light yellowish-brown for cattail-fiber-based webs. Although the inclusion of natural fibers might reduce the mechanical properties of webs, it significantly enhanced water vapor permeability, increased the water contact angle, offered protection against water droplet penetration, and allowed water vapor to pass through the web. Incorporating up to 30% of waste natural fibers met the required tensile strength for protective clothing in a dry state. At the same ratio, the kapok-fiber-based composite web exhibited a higher water contact angle and water vapor permeability compared with cattail fiber, potentially due to the hollow tube structure of kapok fibers. Utilizing waste natural fibers in the production of biocomposites provides eco-friendly alternatives to tackle pollution while innovating functional items. These biocomposites, owing to their renewable and biodegradable nature, emit fewer petroleum-based carbon emissions throughout their life cycle. Future developments aim to reduce the web’s thickness and explore additional properties to meet surgical gown requirements, including resistance to water penetration by impact, hydrostatic pressure, and synthetic blood under continuous liquid contact.

## Figures and Tables

**Table 1:** Antibacterial activity of fibers against *S. aureus* and *K. pneumoniae* bacteria after contact time.

Type of Bacteria	Bacterial Reduction (%)		
	Cattail Fiber	Kapok Fiber	PLA Bicomponent Fiber
*S. aureus*	7.78	16.73	1.41
*K. pneumoniae*	−8.25	−6.98	−7.51

**Table 2:** Thickness values of the composite webs *.

PLA: Natural Fiber Ratio	Thickness (mm)	
	PLA: Cattail Fiber	PLA: Kapok Fiber
100:0	0.2594 ± 0.0328 ^bcdefgh^	0.2594 ± 0.0328 ^bcdefgh^
90:10	0.3436 ± 0.0365 ^adefgh^	0.3798 ± 0.0188 ^aefgh^
80:20	0.3606 ± 0.0211 ^aefgh^	0.3812 ± 0.0160 ^aefgh^
70:30	0.4080 ± 0.0424 ^abefgh^	0.4268 ± 0.0393 ^aefgh^
60:40	0.4686 ± 0.0165 ^abcdfgh^	0.5024 ± 0.0352 ^abcdgh^
50:50	0.5288 ± 0.0190 ^abcde^	0.5648 ± 0.0502 ^abcdh^
40:60	0.5468 ± 0.0340 ^abcde^	0.7020 ± 0.1192 ^abcde^
30:70	0.6286 ± 0.1049 ^abcde^	0.8252 ± 0.1309 ^abcdef^

* Values within the same column with different letters are significantly different at *p* < 0.05. Each value is the mean ± SD of three replicates.

**Table 3:** Whiteness and CIE L*a*b* values of composite webs.

PLA: Natural Fiber Ratio	PLA: Cattail Fiber				PLA: Kapok Fiber			
	WI ^1^	L*	a*	b*	WI ^1^	L*	a*	b*
100:0	86.49	94.71	−0.20	0.08	86.49	94.71	−0.20	0.08
90:10	44.98	87.09	1.71	4.89	48.54	90.12	−0.18	6.31
80:20	32.86	85.35	1.58	6.86	34.28	88.87	1.09	8.13
70:30	13.84	84.00	2.36	7.38	21.77	87.69	1.49	10.07
60:40	−6.86	76.90	2.78	9.74	16.85	86.85	1.81	10.60
50:50	−21.85	74.04	3.84	11.05	7.02	85.68	2.23	11.94
40:60	−61.94	71.31	5.15	16.08	−3.17	84.38	2.46	13.25
30:70	−57.00	70.48	5.29	16.52	−4.38	83.20	2.29	13.47

^1^ WI, whiteness index.

**Table 4:** Mechanical properties of composite webs *.

Sample	PLA: Natural Fiber	Ultimate Load (N)	Young’s Modulus (MPa)	Elongation at Break (%)	Yield Strength (MPa)	Yield Strain (%)
	100:0	47.08 ± 2.01 ^abcdef^	551.76 ± 10.75 ^abcdefg^	8.17 ± 0.64 ^abcdef^	10.62 ± 0.90 ^abcde^	5.99 ± 0.14 ^abcdef^
PLA: cattail fiber	90:10	42.19 ± 3.91 ^abcde^	523.71 ± 6.11 ^abcdefh^	7.90 ± 0.46 ^abcdef^	10.85 ± 0.65 ^abcdef^	5.84 ± 0.07 ^abcde^
	80:20	33.95 ± 0.75 ^abcdegh^	470.29 ± 12.25 ^abcdegh^	4.07 ± 0.24 ^gh^	8.71 ± 0.22 ^abcdefh^	4.79 ± 0.50 ^abcdh^
	70:30	21.18 ± 2.53 ^abcdfgh^	309.72 ± 12.11 ^abcdfgh^	4.33 ± 1.47 ^gh^	4.44 ± 0.80 ^abcfgh^	3.91 ± 0.45 ^adgh^
	60:40	15.10 ± 0.88 ^abcefgh^	279.23 ± 13.57 ^abcefgh^	3.26 ± 0.96 ^gh^	3.70 ± 0.43 ^abcfgh^	2.33 ± 0.97 ^fgh^
	50:50	7.23 ± 0.68 ^defgh^	153.95 ± 13.45 ^abdefgh^	3.68 ± 0.89 ^gh^	1.84 ± 0.08 ^defgh^	2.75 ± 0.40 ^efgh^
	40:60	7.01 ± 0.87 ^defgh^	115.84 ± 8.14 ^cdefgh^	3.36 ± 0.78 ^gh^	1.63 ± 0.22 ^defgh^	2.68 ± 0.71 ^fgh^
	30:70	6.02 ± 0.79 ^defgh^	117.28 ± 10.44 ^cdefgh^	3.64 ± 0.60 ^gh^	1.41 ± 0.22 ^defgh^	2.11 ± 0.52 ^efgh^
	100:0	47.08 ± 2.01 ^abcdef^	551.76 ± 10.75 ^abcdefg^	8.17 ± 0.64 ^abcdfh^	10.62 ± 0.90 ^abcde^	5.99 ± 0.14 ^abcdfg^
PLA: kapok fiber	90:10	45.48 ± 2.85 ^abcde^	451.37 ± 35.69 ^abcdeh^	6.79 ± 0.24 ^acgh^	9.43 ± 1.22 ^abcdeh^	4.37 ± 0.62 ^abcdf^
	80:20	39.88 ± 3.08 ^abcdeh^	408.35 ± 23.61 ^abcdeh^	6.42 ± 0.31 ^acgh^	10.83 ± 0.57 ^abcdef^	4.76 ± 0.65 ^abcdh^
	70:30	22.46 ± 1.58 ^abdfgh^	295.28 ± 48.84 ^abdfgh^	6.55 ± 0.81 ^ach^	7.10 ± 1.52 ^abdfgh^	4.51 ± 0.84 ^aceh^
	60:40	18.13 ± 2.16 ^abefgh^	224.79 ± 35.02 ^abefgh^	3.96 ± 1.46 ^gh^	3.82 ± 0.22 ^abdfgh^	2.67 ± 0.97 ^egh^
	50:50	15.81 ± 0.19 ^abfgh^	170.42 ± 14.75 ^abfgh^	4.45 ± 0.65 ^defgh^	3.85 ± 0.93 ^cefgh^	2.56 ± 0.72 ^eghf^
	40:60	8.44 ± 0.12 ^acdefgh^	129.35 ± 1.83 ^acdefgh^	3.67 ± 0.48 ^defgh^	1.85 ± 0.29 ^cefgh^	2.16 ± 0.74 ^egh^
	30:70	1.59 ± 0.03 ^bcdefgh^	21.73 ± 6.72 ^bcdefgh^	9.77 ± 0.01 ^abcdefg^	0.25 ± 0.01 ^cefgh^	1.87 ± 0.34 ^efgh^

* Values within the same column with different letters are significantly different at *p* < 0.05. Each value represents the mean ± SD of five replicates.

## Data Availability

The data presented in this study are available on request from the corresponding author.
